# Differential epigenetic regulation between the alternative promoters, *PRDM1α* and *PRDM1β*, of the tumour suppressor gene *PRDM1* in human multiple myeloma cells

**DOI:** 10.1038/s41598-020-72946-z

**Published:** 2020-09-28

**Authors:** Raquel Romero-García, Laura Gómez-Jaramillo, Rosa María Mateos, Gema Jiménez-Gómez, Nuria Pedreño-Horrillo, Esther Foncubierta, Juan Francisco Rodríguez-Gutiérrez, Sebastián Garzón, Francisco Mora-López, Carmen Rodríguez, Luis M. Valor, Antonio Campos-Caro

**Affiliations:** 1 Unidad de Investigación, Hospital Universitario Puerta del Mar, Avenida Ana de Viya 21, 11009 Cádiz, Spain; 2 Instituto de Investigación e Innovación en Ciencias Biomédicas de Cádiz (INiBICA), Avenida Ana de Viya 21, 11009 Cádiz, Spain; 3 Área Bioquímica y Biología Molecular, Dpto. Biomedicina Biotecnología y Salud Pública, Universidad de Cádiz, 11510 Cádiz, Spain; 4 Unidad de Hematología, Hospital Universitario de Jerez, Ronda de Circunvalación s/n, 11407 Jerez de La Frontera, Spain; 5 Servicio de Inmunología, UGC de Hematología, Inmunología y Genética, Hospital Universitario Puerta del Mar, 11009 Cádiz, Spain; 6 Área Genética, Dpto. Biomedicina Biotecnología y Salud Pública, Universidad de Cádiz, 11510 Cádiz, Spain

**Keywords:** Immunology, Molecular biology, Oncology

## Abstract

Multiple myeloma (MM) is a B-cell neoplasm that is characterized by the accumulation of malignant plasma cells in the bone marrow. The transcription factor PRDM1 is a master regulator of plasma cell development and is considered to be an oncosuppressor in several lymphoid neoplasms. The PRDM1β isoform is an alternative promoter of the PRDM1 gene that may interfere with the normal role of the PRDM1α isoform. To explain the induction of the PRDM1β isoform in MM and to offer potential therapeutic strategies to modulate its expression, we characterized the *cis* regulatory elements and epigenetic status of its promoter. We observed unexpected patterns of hypermethylation and hypomethylation at the PRDM1α and PRDM1β promoters, respectively, and prominent H3K4me1 and H3K9me2 enrichment at the PRDM1β promoter in non-expressing cell lines compared to PRDM1β-expressing cell lines. After treatment with drugs that inhibit DNA methylation, we were able to modify the activity of the PRDM1β promoter but not that of the PRDM1α promoter. Epigenetic drugs may offer the ability to control the expression of the PRDM1α/PRDM1β promoters as components of novel therapeutic approaches.

## Introduction

PRDM1 (PR domain zinc finger 1) is a transcriptional factor that was initially identified as a 98-kDa DNA-binding repressor of β-interferon gene expression^1^, which is broadly known as BLIMP1 (B lymphocyte-induced maturation protein-1) because of its key role in the terminal differentiation of B cells towards plasma cells (PCs)^2^. Moreover, PRDM1 expression is required for the formation of Ig-secreting cells^3^ and the maintenance of long-lived PCs in the bone marrow^4^. However, PRDM1 has been subsequently shown to play critical regulatory roles in the differentiation, activation and homeostasis of other cell types^5,6^, development-related pathways in embryos and homeostasis in adults^7,8^.

The N-terminal half of the protein contains the PR domain, a subclass of the Su(var)39-1, Enhancer of Zeste, Trithorax (SET) family of methyltransferase domains that methylate histone H3 on lysine 9 (H3K9)^9^, which is a feature of the proteins belonging to this family. However, in contrast to other members of this family, no methyltransferase activity has been demonstrated by PRDM1. Therefore, PRDM1 achieves its repressor activity by acting as a corepressor with other proteins, including histone deacetylases^10^, G9a lysine methyltransferase^11^, protein arginine methyltransferase 5 (PRMT5)^12^, lysine-specific demethylase 1 (LDS1)^13^ and Groucho family proteins^14^. PRDM1 has been proposed to have modular control of gene expression^15^ with the ability to regulate different subsets of genes depending on the domains and proteins with which it interacts^10^.

The human *PRDM1* gene contains seven coding exons and presents two alternative promoters capable of generating the two transcript isoforms: PRDM1α (NM_001198) and PRDM1β (AY198415). PRDM1β is generated through a promoter and an additional exon (exon-1β) located in intron 3 adjacent to exon 4. Exon-1β contains a short 5′ UTR and encodes only 3 aa (MEK) before joining exons 4 to 7. Therefore, PRDM1β is a shorter isoform (691 aa) than PRDM1α (825 aa), lacking 134 aa of the N-terminus (which comprises a small acidic region and a portion of the PR regulatory domain in PRDM1α). The PRDM1β isoform exhibits significantly impaired repressor activity in multiple target genes^16^, similar to PR-related isoforms of other PRDMs, such as PRDM2^17^, PRDM3^18^ and PRDM16^19^. The presence of the shorter isoform with a hypomorphic function may result in an imbalance in a “yin-yang” fashion between the two isoforms and may be critical for tumorigenesis^20^. This PRDM1α/PRDM1β imbalance can be the result of inactivating PRDM1α by means of gene mutations^21–23^ or promoter hypermethylation^24,25^ and by the activation/overexpression of PRDM1β^26,27^. In the latter case, an increase in the PRDM1β isoform at the mRNA level has been detected in both myeloma cell-derived lines and multiple myeloma samples^16,28^ and in lymphomas (diffuse large B-cell lymphoma^27,29^, T-cell lymphoma^30^ and EBV-associated lymphomas^26^). While the *PRDM1α* promoter has been extensively studied in mice^31–34^ and humans^35–37^, few studies have examined the *PRDM1β* promoter, and they were focused on its methylation status in lymphomas^25,26,29^. To date, there are no reports of its role in multiple myeloma.

As PRDM1β is a truncated isoform considered to compete with the full-length PRDM1α isoform and because its overexpression in myeloma cells may be functionally relevant in tumorigenesis, we studied the regulation of the human *PRDM1β* promoter as a potential therapeutic target. To this end, we took two parallel approaches: (i) characterizing the *cis*-sequences implicated in its transcriptional expression and (ii) by analysing its epigenetic status and comparing it to PRDM1α in human myeloma cells.

## Results

### Overexpression of PRDM1α but not PRDM1β induces the apoptosis of U266 cells

**Figure 1:**
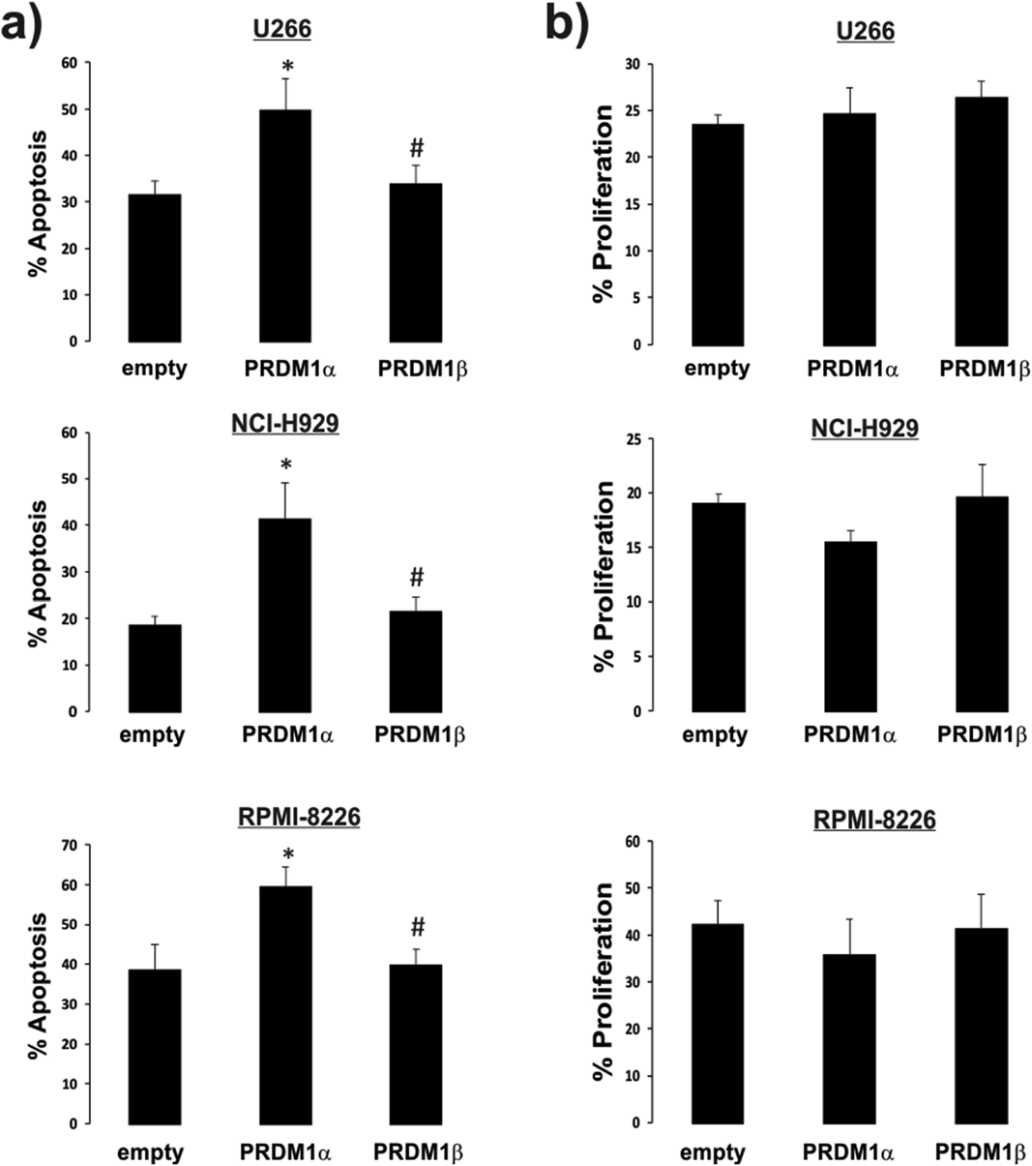
Apoptosis was induced by overexpressing the PRDM1 isoforms. U266, NCI-H929 and RPMI-8226 cells were transfected with an empty pIRES2-GFP vector or with the pIRES2-EGFP-PRDM1α or pIRES2-EGFP-PRDM1β expression vectors. Positively transfected U266 cells (EGFP^+^ cells) were analysed by flow cytometry after labelling the cells with Annexin V-APC for apoptosis **a** or with Click-iT plus EdU Alexa 647 for proliferation **b**. Results indicate the percentage of apoptotic or proliferating cells based on the empty vector used as a control. The data represent the means ± SEM (n = 4), Mann–Whitney U test, * and ^#^*p* < 0.05 compared to the control and PRDM1α, respectively.

Impaired PRDM1β ability to repress important genes implicated in the cell cycle (e.g., the *MYC* gene) has been described^16^. Moreover, we demonstrated that PRDM1β isoform expression is augmented in multiple myeloma cells isolated from patient samples^28^, which was confirmed and correlated with the disease status of myeloma patients in a subsequent study^38^. However, the effect of PRDM1α and/or PRDM1β overexpression on proliferation and apoptosis has not been previously tested in myeloma cells. To this end, U266, NCI-H929 and RPMI-8226 cells were transfected with vectors expressing the PRDM1α and PRDM1β isoforms. Figure 1A shows that the overexpression of PRDM1α, but not PRDM1β, increased the apoptosis of the U266-transfected cells. However, neither PRDM1α nor PRDM1β overexpression affected the proliferation rate (Fig. 1B). Considering these observations and because it is virtually impossible to design specific knockdown assays for both isoforms, as their cDNA coding sequence only differs in 3 codons, we reasoned that the lower PRDM1α/PRDM1β ratio in myeloma cells, compared to that in normal cells, caused the accumulation of malignant cells due to inhibited apoptosis, not an increase in cell proliferation. More sophisticated manipulations would determine whether this reduction in apoptotic events contributes to the development of the myeloma. Therefore, we decided to analyse the unexplored transcriptional regulation of the PRDM1β isoform and the effect of epigenetic regulators on the expression of both isoforms in myeloma cells.

### Identification of a repressive regulatory element of PRDM1β expression

**Figure 2:**
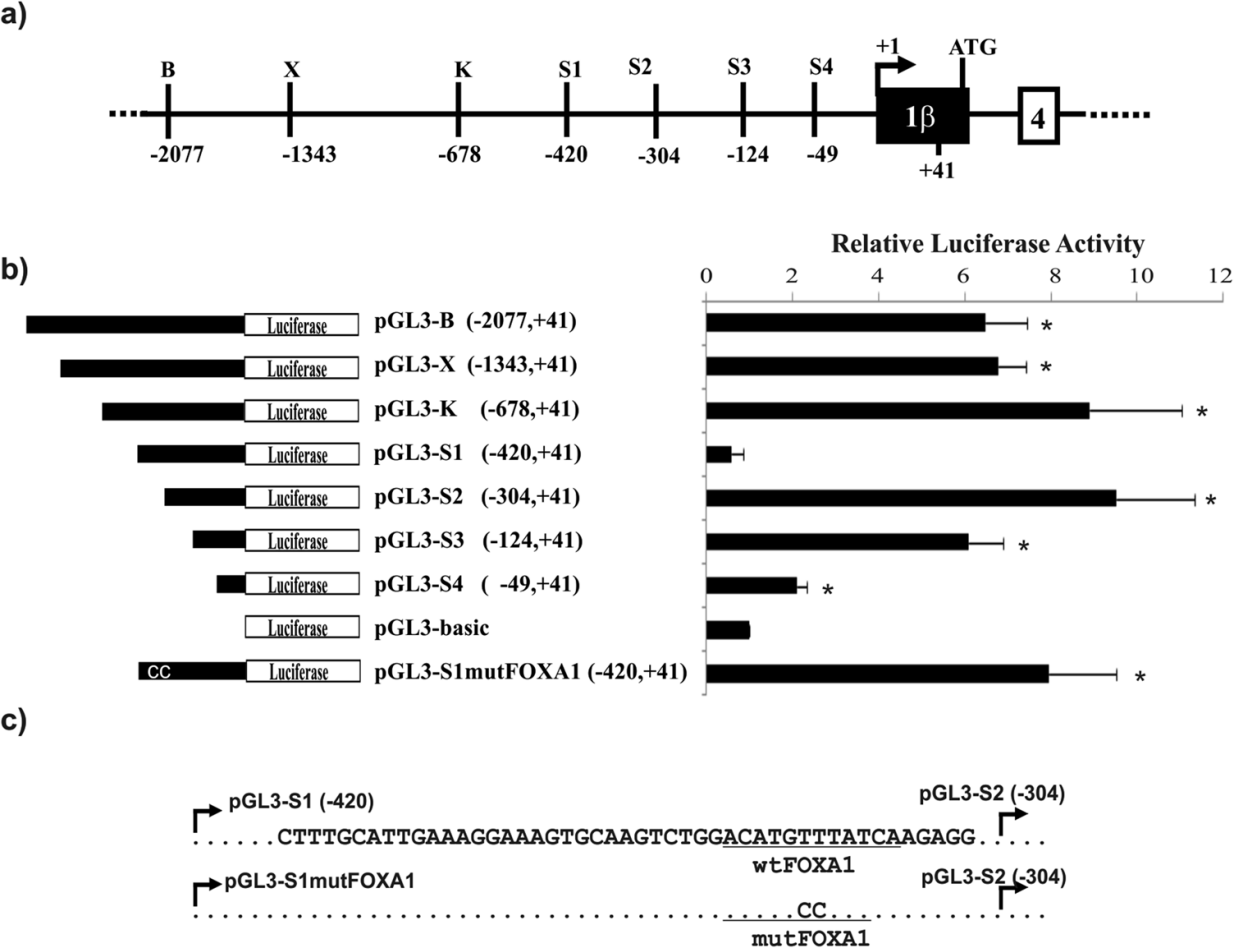
Dissecting the regulatory regions of the human *PRDM1β* promoter. **a** Representative scheme of the relative positions used in the reporter plasmid constructs. Positions are relative to the TSS according to the sequence AY198415. The first exon in the *PRDM1β* isoform is shown as a filled black box, and exon 4 (in isoform *PRDM1α* but common to both isoforms) is indicated as an empty box. **b** Relative luciferase activity levels of the *PRDM1β* promoter constructs. Human U266 cells were transiently transfected with the indicated reporter constructs. The fold change in luciferase activity was normalized to the signal obtained with a pGL3-basic vector (without any regulatory insert). The results represent the mean ± SEM of at least five experiments with triplicate samples. Significant induction (*p* < 0.05) is indicated by an asterisk. The pGL3-S1 mutFOXA1 construct was identical to pGL3-S1, except for a point mutation in the FOXA1-binding motif (**c**).

To understand the regulatory mechanisms of transcriptional regulation for both isoforms and considering that the upstream regulatory elements and factors controlling the transcription of the human *PRDM1β* promoter have not been previously described, we decided to identify the putative* cis* regulatory elements that are critical for the basal expression of *PRDM1β*. To this end, we cloned and analysed a genomic fragment ~ 2 Kbp upstream of the transcription start site (TSS) in the *PRDM1 gene* extracted from GenBank, sequence accession number AL022067 (Fig. 2A). To evaluate the transcriptional mechanisms, the fragment was gradually truncated to create a series of 5′-end-deletion constructs cloned from positions − 2077 to + 41 (pGL3-BamHI), − 1343 to + 41 (pGL3-XbaI), − 678 to + 41 (pGL3-KpnI) − 420 to + 41 (pGL3-S1), − 304 to + 41 (pGL3-S2), − 124 to + 41 (pGL3-S3), and − 49 to + 41 (pGL3-S4) (numbered from the TSS according to the sequence AY198415^16^). All inserts were cloned upstream of the firefly luciferase reporter gene in a pGL3-basic vector (Fig. 2B). These constructs were transiently transfected into U266 myeloma cells, which expresses endogenous PRDM1^16,28^. As shown in Fig. 2B, the most striking finding was the complete loss of promoter activity observed with the pGL3-S1 construct but not with the pGL3-S2 construct. The transcriptional recovery observed by the removal of the segment − 420/− 304 suggested that a *cis-*element with repressive actions on *PRDM1β* was present in this region (Fig. 2B). Similar results were obtained using the PRDM1β-expressing NCI-H929 cell line (Supplementary Fig. S1). RPMI-8226 was not used in this set of experiments because, as explained below, it did not express the PRDM1β isoform. After a sequence analysis of the S1-S2 fragments using Alibaba2 software based on the TRANSFAC database^39^, we identified several motifs as potential candidates for *cis-*acting elements (Supplementary Fig. S2). In addition to general transcription factors such as GATA, SP1, and OCT1, we observed a possible binding site for the transcription factor FOXA1 (also known as HNF3A), which is implicated in the establishment of lineage-specific transcriptional enhancers and programmes^40^. However, this DNA motif is shared among the FOX family members, and therefore, we referred to this sequence indistinctly as the FOX or FOXA1 motif hereafter. Furthermore, this binding site matched one of the two half-sites in the consensus sequence for TP53 binding previously described by another group^41^ (Fig. 2A). Therefore, we decided to mutate this DNA motif as shown in Fig. 2C. Using luciferase assays, we confirmed the repressive role of this element, as the corresponding mutated FOXA1-site construct, which is homologous to the pGL3-S1 construct (pGL3-S1mutFOXA1), recovered the promoter activity to a level similar to that of the pGL3-S2 and pGL3-S3 constructs (Fig. 2B).

### Repressive elements of the PRDM1β promoter do not bind TP53

**Figure 3:**
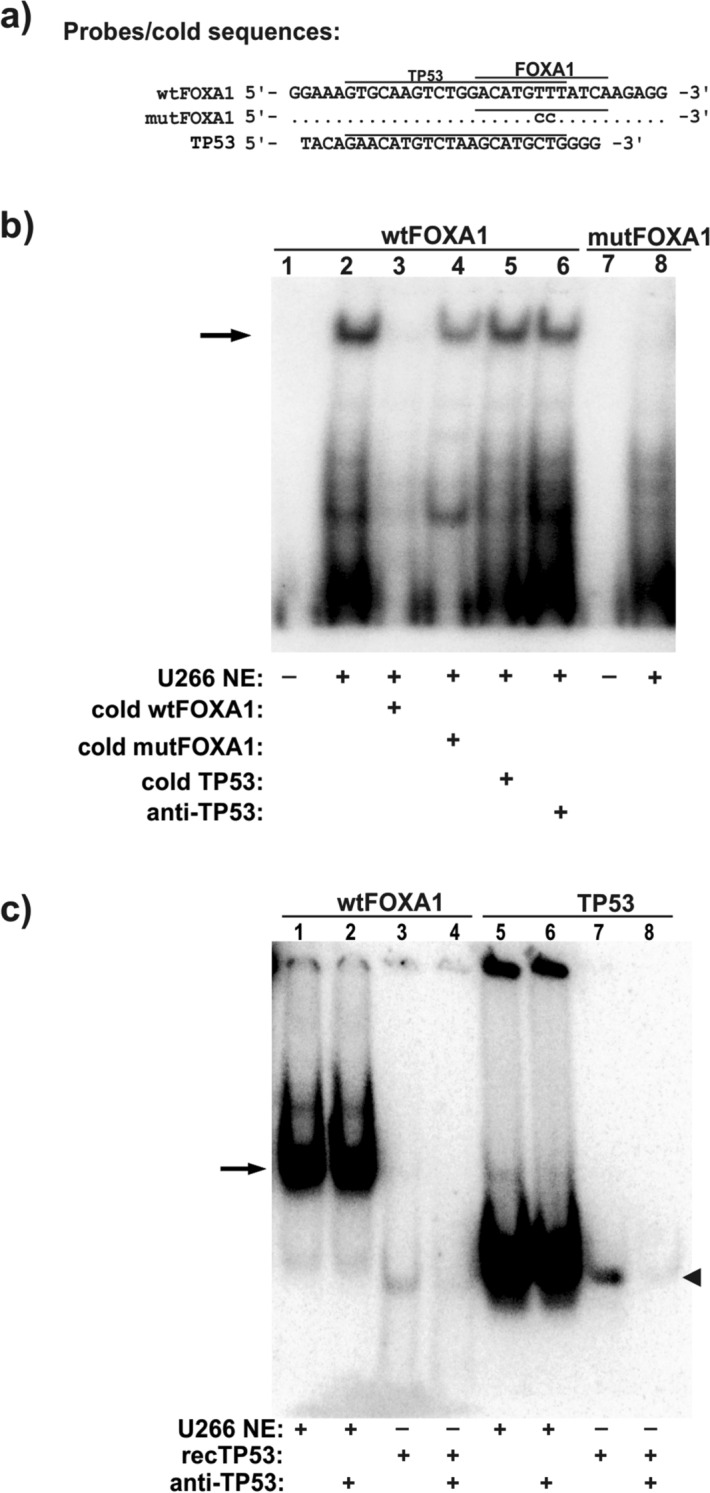
The repressive element of the human *PRDM1β* promoter binds nuclear proteins but not FOXA1 or TP53. **a** Sequences used as probes or cold competitor fragments (only the DNA strand + is represented). Putative wtFOXA1-and TP53-binding sites are indicated. Sequences in the mutated FOXA1 fragment and a sequence used as a putative TP53 (Panomics, Fremont, CA) are also shown. **b** EMSA with NE from the U266 cell line and wtFOXA1 or mutFOXA1 probes. Lane 1, probe without NE; lane 2, probe incubated with NE; lanes 3, 4 and 5 correspond to the competition experiments using cold fragments (100 × excess) with the same sequence as the labelled probe (wtFOXA1), the mutated version (mutFOXA1) or the TP53 binding site fragment, respectively; lane 6, supershift assay performed using the same conditions as indicated for lane 2 with the addition of an anti-human TP53 specific antibody (Sigma, P6874); lanes 7 and 8, mutated probe FOXA1 without or with U266 NE, respectively. The main retarded band is indicated by an arrow. **c** Lanes 1 and 3, EMSA using a wtFOXA1 probe with U266 NE showing the positions of the main retarded band (indicated by an arrow) and the shifted band observed using recombinant human TP53 protein instead of NE (indicated by an arrowhead). Lanes 2 and 4 show the same conditions as lanes 1 and 3 with the addition of an anti-TP53 antibody for the supershift assays. Lanes 5–8, EMSAs and similar to lanes 1–4 but with the TP53 probe instead of the FOXA1 probe.

Next, we further explored the potential role of the FOX-binding site in *PRDM1β* gene regulation. Thus, we prepared two probes from different regions of the S1–S2 construct that contained either the putative wild-type FOX-binding site (wtFOXA1) or the mutated binding site (mutFOXA1) (see sequences in Fig. 3A). The labelled probes were used in EMSAs with nuclear extract (NE) from U266 cells. As shown in Fig. 3B, different retarded band patterns were observed for the wtFOXA1 (lanes 1–2) and mutFOXA1 (lanes 7–8) probes, indicating that one or more factors were able to bind the wild-type but not the mutated sequences. Then, we investigated whether some of these bands were retarded because of TP53 binding to the wild-type probe. Thus, competition assays were conducted using wtFOXA1 (lane 3), mutFOXA1 (lane 4) and a double-stranded oligonucleotide containing the consensus binding site of TP53 (lane 5) as cold fragments (Fig. 3A). As expected, the retarded bands were abrogated with the wild-type version of the competitor probe, whereas the mutFOXA1 and TP53 cold fragments were unable to effectively compete with the labelled probe, demonstrating the specificity of the protein binding to the FOXA1 motif; in addition, the unidentified factor did not bind the putative TP53-binding site. This negative result was confirmed in a supershift experiment using two different antibodies against human TP53 (Fig. 3A, lane 6 and Supplementary Fig. S3), in which no changes in the band pattern were observed. Other related proteins (TP63 and TP73) were not detected (data not shown). Moreover, the anti-FOXA1 antibody did not lead to any supershift or eliminated retarded bands (Supplementary Fig. S3), indicating that these bands did not contain any of these transcription factors. To further corroborate that the retarded band corresponded to a factor other than the TP53 protein, additional EMSAs were performed using the wtFOXA1 and TP53 probes with U266 NE and recombinant TP53 protein. Figure 3C shows the different patterns of the bands obtained with the U266 NE and with recombinant TP53 using the wtFOXA1 probe (lane 1 vs. 3). After adding an anti-TP53 antibody to the supershift assays, only the retarded band corresponding to TP53 was faded (lane 2 vs. 4). Under the same experimental conditions using a putative TP53 probe, the same retarded band was observed regardless of whether U266 NE or recombinant TP53 was used (lanes 5–8). This assay also confirmed the expression of endogenous TP53 in the U266 cells. These results indicated that the retarded band observed with the wtFOXA1 probe was not a consequence of TP53 binding to the probe. Furthermore, HeLa cells, which do not express TP53, were transfected with constructs containing the human TP53 cDNA, in either the wild-type form (wtTP53) or a mutated dominant negative form (TP53DN), to examine their effects on the regulation of the *PRDM1* promoters. Thus, the *PRDM1α* and *PRDM1β* transcript levels in the HeLa cells transfected with wtTP53, TP53DN or an empty vector were analysed by RT-qPCR, and no difference was observed between them (Supplementary Fig. S4A), suggesting that, at least in this cell line, TP53 did not regulate *PRDM1* gene transcription. Exogenous TP53 and TP53DN protein expression was confirmed by western blotting (Supplementary Fig. S4B). Therefore, in contrast to what has been described in cell line-derived colon cancer^41^, TP53 does not seem to have a role in *PRDM1* transcriptional regulation. Future proteomic approaches should be applied to determine the identity of the regulatory factor that binds the repressive *cis*-element of the *PRDM1β* promoter.

### Promoter methylation status shows a higher correlation with PRDM1β expression than with PRDM1α expression

**Figure 4:**
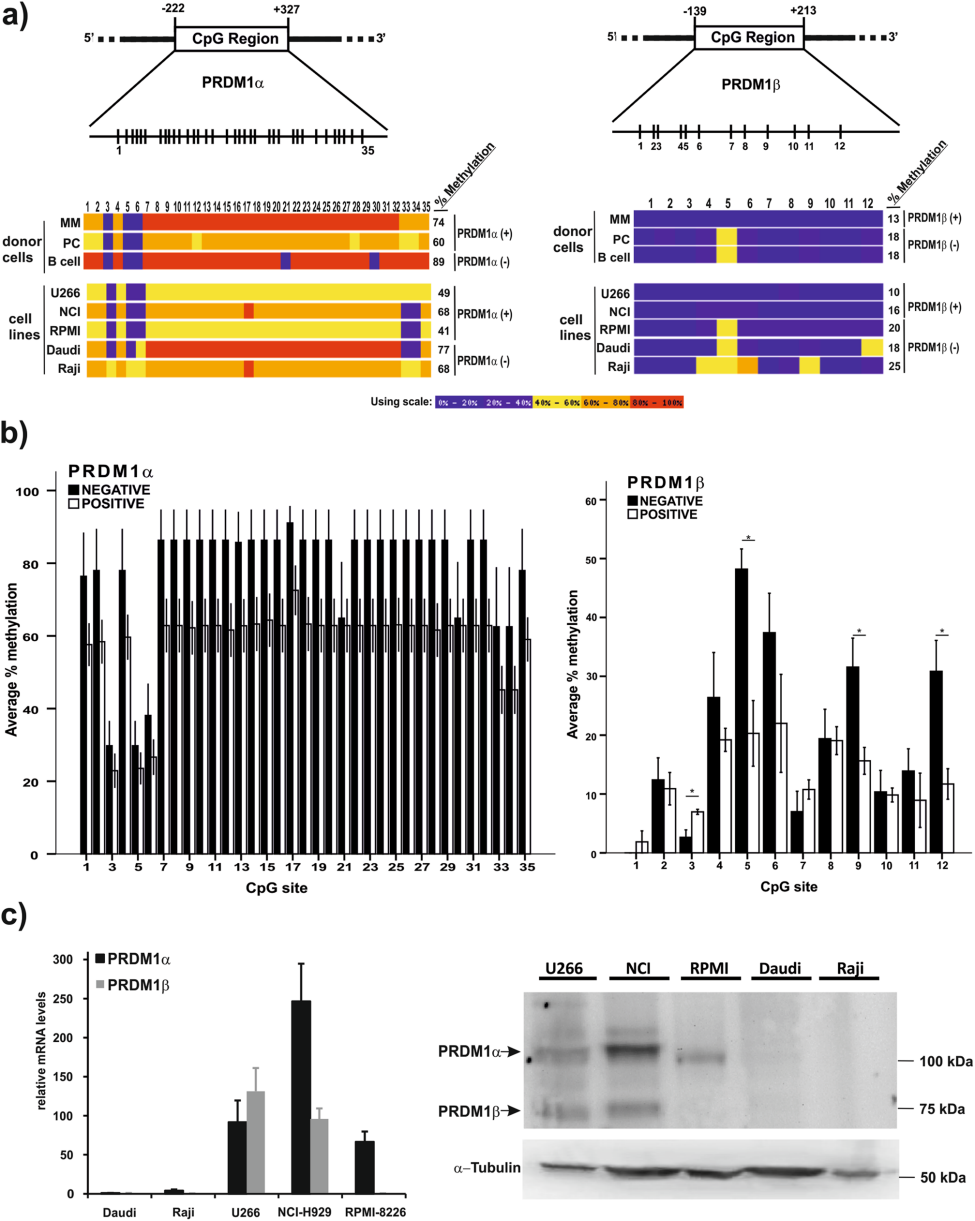
Methylation status of the *PRDM1α* and *PRDM1β* promoters in myeloma cell lines, normal and tumour B cells and PC subsets. **a** Representative CpG island positions of the 35 and 12 CpG methylation sites analysed by bisulfite sequencing of the *PRDM1α* and *PRDM1β* promoters, respectively. BISMA program^59^ results are shown in a colour code indicating the percentage of methylation at each CpG position and the cell origin of the clones analysed: for *PRDM1α*: MM-PCs from 7 patients (57 clones), PCs from 4 donors (32 clones), B cells from 3 donors (31 clones), U266 cells (14 clones), RPMI-8226 cells (clones 10), Raji cells (clones 10), NCI-H929 cells (clones 12), and Daudi cells (clones 8); for *PRDM1β*: MM-PCs from 7 patients (71 clones), PCs from 4 donors (47 clones), B cells from 3 donors (41 clones), U266 cells (17 clones), RPMI-8226 cells (clones 15), Raji cells (clones 16), NCI-H929 cells (clones 13), and Daudi cells (clones 16). All sequencing analyses of both promoters were performed using the same bisulfite conversion sample. Expression (or the lack of expression) of the PRDM1α and PRDM1β isoforms in the tested cell types is indicated as PRDM1α/β positive or negative. **b** Total average methylation percentage at each CpG site in both promoters after grouping all cells, based on whether the cells express the *PRDM1α* or *PRDM1β* transcript, is shown^59^. (*) Indicates *p* < 0.05. **c** Expression of the *PRDM1α* and *PRDM1β* transcript isoforms in the indicated cell lines was determined by RT-qPCR assays (left panel) (means ± SEM, n = 3) and western blot analysis (right panel) using 80 µg of total protein per lane and an anti-PRDM1 antibody recognizing both isoforms (Santa Cruz, sc-66015). α-Tubulin was used as a loading control. A representative experiment is shown; the arrows indicate both isoforms.

In a previous report, we showed that the expression level ratio of PRDM1β/PRDM1α was altered in plasma cells from bone marrow samples of multiple myeloma patients^28^. Due to the relevant role of epigenetics in cancer and tumorigenesis, we investigated DNA methylation patterns in a more natural environment (as opposed to the in vitro assays described above) in our cellular preparations. To correlate their respective expression levels to the methylation status of their promoters, we examined the methylation pattern in both promoters within the same samples isolated from several cell lines and primary cells from healthy donors and patients (naïve B-cells, PCs and MM-PCs). In total, 35 and 12 CpG dinucleotides in the *PRDM1α* and *PRDM1β* promoters, respectively, were selected as potentially methylable for bisulfite sequencing analysis (Fig. 4A). The *PRDM1α* promoter was hypermethylated compared to the *PRDM1β* promoter in all tested cell types. Thus, cells well known to be negative for PRDM1α expression, such as the Raji and Daudi lymphoma cell lines, and normal B cells, showed high methylation levels, ~ 68%, ~ 77% and ~ 89%, respectively (Fig. 4A). Strikingly, in the myeloma cell lines positive for PRDM1α expression (RPMI-8226, U266 and NCI-H929, Fig. 4C), the methylation status was also relatively elevated (~ 41%, ~ 49% and ~ 68%, respectively). A similar pattern was observed in the *PRDM1α* promoter in the PCs from healthy donors (~ 59%) and cells from patients diagnosed with multiple myeloma (MM-PCs, ~ 74%). Therefore, the hypermethylation of the *PRDM1α* proximal promoter was apparently irrelevant for PRDM1α expression. Nevertheless, a more informative methylation pattern was observed in the *PRDM1β* promoter based on a comparison of PRDM1β-expressing and non-expressing cells (Fig. 4B). Thus, as shown in Fig. 4A, PRDM1β-positive cells, such as U266 cells (~ 10%), NCI cells (~ 16%) and MM-PCs (~ 13%), presented a relatively low methylation level compared to that observed in the following PRDM1β-negative cells: Raji cells (~ 25%), Daudi cells (~ 18%), normal PCs (~ 18%) and normal B cells (~ 18%). Surprisingly, the classical myeloma cell line RPMI-8226 presented > 20% of the CpG islands methylated, and PRDM1β was not expressed either as a transcript or protein (Fig. 4C). Deeper analysis performed to identify possible methylable CpG positions as determinants of PRDM1 expression revealed no significant differences in the *PRDM1α* promoter (Fig. 4A,B, and Supplementary Fig. S5). However, CpG-5, CpG-9 and CpG-12 in the *PRDM1β* promoter were significantly more methylated in non-expressing cells than in expressing cells, and the same trend was also observed for CpG-4 and CpG-6.

### PRDM1β, but not PRDM1α, is regulated by the 5-aza-dC treatment of myeloma cells

**Figure 5:**
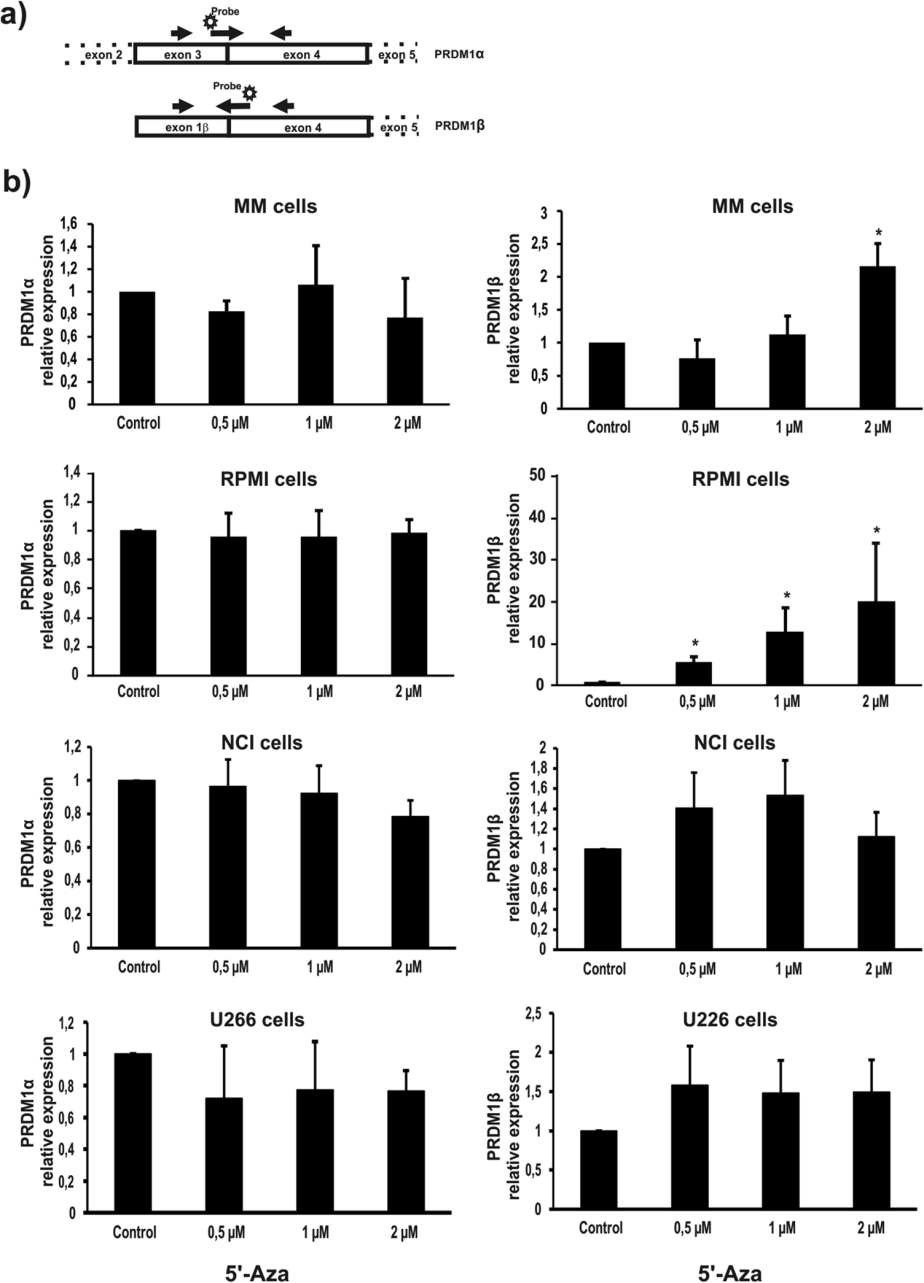
*PRDM1α* and *PRDM1β* expression after 5-aza-dC treatment. **a** Schematic probe and primer pair locations on cDNA used for detecting the two transcripts (the sequences are listed in Table 1). **b** MM-PCs and RPMI-8226, NCI-H929 and U266 cells were treated with the DNA methyltransferase inhibitor 5-aza-dC (0, 0.5, 1 and 2 µM) for 2 days. RT-qPCR assays of PRDM1α and PRDM1β transcript expression were performed. The mean ± SEM of n = 3 independent experiments are shown. (*) Indicates *p* < 0.05.

The results revealed that, in general, the methylation levels of the *PRDM1α* promoter are higher than those of the *PRDM1β* promoter regardless of the expression of *PRDM1* transcripts. To corroborate the observation suggesting that promoter methylation status can preferentially affect the transcription of *PRDM1β* relative to that of *PRDM1α*, an RT-qPCR assay was designed in myeloma cells treated with the DNA methyltransferase inhibitor 5-aza-dC (Fig. 5A). The results showed that treatment with 5-aza-dC increased the expression of the *PRDM1β* transcript isoform but had no apparent effect on the *PRDM1α* transcript level (Fig. 5B). These results supported the observation that promoter methylation is relevant for the expression of *PRDM1β* but not *PRDM1α,* as the latter isoform is present in all cells, even those with a hypermethylated promoter.

### Histone marks associated with PRDM1 gene transcription

**Figure 6:**
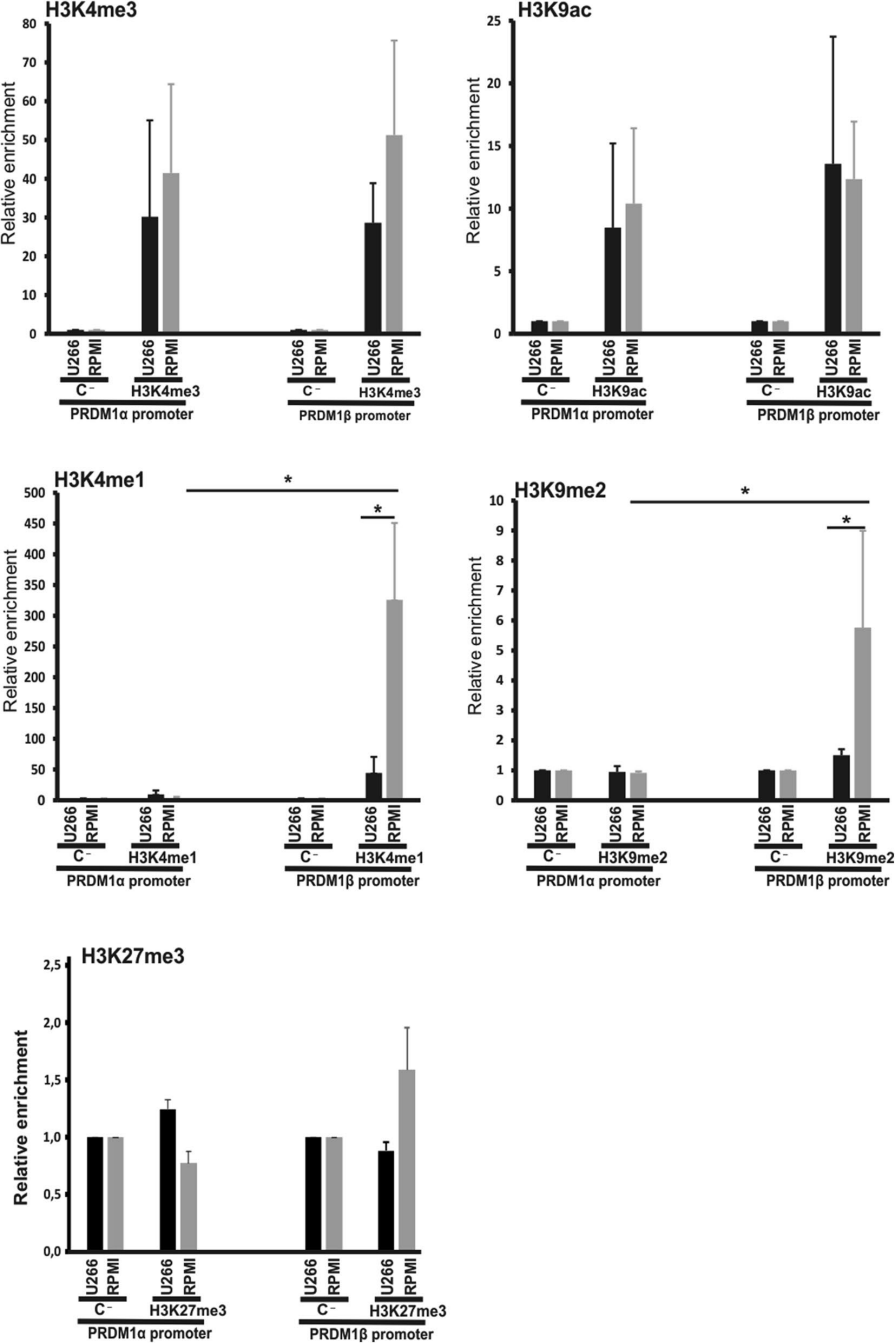
Histone marks at the *PRDM1α* and *PRDM1β* promoters in U266 and RPMI-8226 cells. ChIP assays of two MM cell lines, i.e., U266 (positive for PRDM1α and PRDM1β) and RPMI-8226 (positive only for PRDM1α), using antibodies against mono- or tri-methylated histone H3-Lys4 (H3K4me1 or H3K4me3) or di-methylated or acetylated H3-Lys9 (H3K9me2 or H3K9ac). The samples were analysed by RT-qPCR. The relative levels of enrichment compared to the controls are shown as the means ± SEM; n = 4 independent experiments. (*) Indicates p < 0.05. Normal rabbit serum was used as a negative control (C-).

In addition to CpG methylation, we examined additional epigenetic mechanisms, such as histone posttranslational modification, as potential contributors to *PRDM1* gene regulation. Thus, we obtained cross-linked chromatin from U266 cells (expressing both PRDM1α and PRDM1β isoforms) and RPMI-8226 cells (expressing only the PRDM1α isoform). Quantitative PCR-based ChIP assays with antibodies against mono- or tri-methylated histone H3 Lys4 (H3K4me1 or H3K4me3), di-methylated H3 Lys9 (H3K9me2) and acetylated H3 Lys9 (H3K9ac) revealed some differences in the histones deposited on the *PRDM1α and PRDM1β* promoters in the U266 and RPMI-8226 cells (Fig. 6). The levels of H3K4me1 (a histone mark associated with enhancer regions but associated with gene repression when located at promoters^42^) and H3K9me2 (also associated with transcriptional repression) were relatively higher on the *PRDM1β* promoter in the RPMI cells than on the promoter in the U266 cells, which is consistent with the lack of expression in the former cell line. In agreement with the latter result, the RPMI cells also showed greater enrichment of H3K27me3 (although the difference was not significant). No differences in these histone marks were observed in the *PRDM1α* promoter. In contrast, the H3K4me3 and H3K9ac levels (both associated with active transcription) were equivalent in both promoters in both cell lines. Overall, the ChIP results suggested that H3K9me2 and/or H3K4me1 were associated with the repression of *PRDM1β*, but not *PRDM1α*, in the RPMI cells. In the U266 cells, which exhibited a predominance of active-associated marks, such as H3K4me3 and H3K9ac, both PRDM1α and PRDM1β proteins were expressed.

## Discussion

The transcriptional activity of PRDM1 is essential for the differentiation and maintenance of long-lived plasma cells^3,4^, whose downregulation has been associated with several types of cancer. Therefore, *PRDM1* is considered a tumour suppressor gene^23,43–47^. The two major isoforms, PRDM1α and PRDM1β, are expressed in myeloma cells, while only the PRDM1α isoform is expressed in non-tumorigenic plasma cells^11,28^. PRDM1β is a truncated isoform that is through to compete with the full-length isoform PRDM1α, which is overexpressed in myeloma cells, leading to the hypothesis that PRDM1β may be functionally relevant in myeloma cells. Nevertheless, there are few studies linking the expression of PRDM1β to myeloma^16,28,38^. In this study, we tested, for the first time, the effect of the overexpression of PRDM1α and PRDM1β isoforms in myeloma cells. We observed that PRDM1α, but not PRDM1β, induced increased apoptosis without apparently affecting cell proliferation in myeloma. These observations led us to consider an alternative explanation for the maintenance of myeloma cells: Rather than an increase in proliferation, expression of the PRDM1β isoform may inhibit apoptosis. However, some contradictory results were obtained in other studies in which PRDM1α altered the proliferation of HCT116^41^ and SW620^47^ cells but not RKO colon cancer cells^48^, and ectopic expression of PRDM1 in a chicken fibroblast cell line inhibited cell proliferation^49^. Depending on the cellular context, further studies on the mechanisms of action of the PRDM1 isoforms will be important for understanding their roles in the regulation of apoptosis, cell proliferation and the cell cycle.

In the present study, luciferase promoter analysis identified a putative *cis-*element in the promoter region -304 to -420 relative to the TSS. The in silico analysis identified a possible binding site for the FOX transcription factors in positions -345 to -334 within that region. Moreover, this site partially overlapped with a previously proposed DNA binding motif for TP53^41^; in the previous work, the authors described a transcriptional regulation loop through which PRDM1 is activated by TP53, which is then repressed by PRDM1. However, we were unable to reproduce TP53 involvement in the regulation of PRDM1 in the EMSAs, an in vitro assay in which proteins bind DNA motifs solely by sequence affinity, or in the overexpression experiments, in which we did not observe any increase/decrease in *PRDM1α* or *PRDM1β* levels in HeLa or U266 cell cultures with western blotting or RT-qPCR (Supplementary Fig. S3 and data not shown, respectively). This lack of reproducibility may be due to, at least, two reasons: (1) the PRDM1/TP53 autoregulatory loop may be dependent on the cell line studied (HTC116 cells in the case of the original publication)^41^, and (2) the previous study referred to PRDM1 activation or repression without clearly distinguishing between the two isoforms PRDM1α and PRDM1β, despite the identification of the TP53 binding site in the *PRDM1β* promoter. However, we failed to identify a transcription factor bound to this promoter in the supershift assays (TP53, Fig. 3 and Supplementary Fig. S2) or in a preliminary mass spectrometry analysis (not shown).

In addition, the previous experiments were based on exogenous DNA sequences that did not properly reproduce the cellular chromatin context. Here, we report, for the first time, the methylation status of alternative promoters of the *PRDM1* gene from isolated myeloma cells. We found that the *PRDM1α* promoter was relatively hypermethylated (range 40–90%) in all the cell lines tested regardless of the expression of the *PRDM1α* transcript or protein. However, the *PRDM1β* promoter generally presented with a much lower methylation level (10–25%) with significant differences in the methylation of specific CpG positions in the cells expressing and not expressing *PRDM1β*. CpG-5, CpG-9 and CpG-12 clearly had greater methylation enrichment in the PRDM1β-negative cells than in PRDM1β-positive cells. No difference in the methylation of any specific CpG position was observed in the *PRDM1α* promoter. In summary, the following conclusions are drawn based on our methylation analysis. First, the overall level of methylation at the *PRDM1α* promoter does not determine its expression in all cells tested; for instance, the Raji cell line (~ 68% methylated) does not express PRDM1α, while the NCI-H929 cell line (~ 68%) and MM cells (~ 74%) both highly express PRDM1α. Second, even though the global methylation level of the *PRDM1β* promoter (< 25%) is much lower than that of the *PRDM1α* promoter (> 40%), the methylation level does not appear to be decisive of the relative expression levels of both isoforms, although one or both promoters may be activated by 5-aza-dC (this article and previous works^24,25,27^). In addition to methylation, other elements, such as specific transcription factors and miRNAs, may be critical for the imbalanced expression of PRDM1α/PRDM1β in tumours^50,51^. Nevertheless, differing results of the methylation status of the *PRDM1α* promoter have been reported. The hypermethylation of the *PRDM1α* promoter in EBV^+^ BL cell lines, including the Daudi cell line, which is hypermethylated as described in Fig. 4A, is associated with low PRDM1 expression^25^. Nonetheless, in contrast to our work, the authors observed only trace or no methylation in normal B-cell subsets (i.e., GC cells and memory and naïve B cells), although it is widely reported that these cell subsets do not express PRDM1α, which is a marker of differentiated PCs. In other reports, the low *PRDM1α* transcript levels were not the result of promoter hypermethylation in diffuse large B-cell lymphoma^51^ or chronic lymphocytic leukaemia^52^, but the opposite results have also been reported in NK-cell malignancies^24,53^. In conclusion, the general lack of consistency between promoter methylation and the gene expression of *PRDM1α* in previous works does not support a relevant role for CpG methylation in the regulation of this isoform. This conclusion is supported by the results of the cell culture treatment with the methyltransferase inhibitor 5-aza-dC, which significantly increased the expression of *PRDM1β* but not *PRDM1α* (Fig. 5).

Another important finding in this study is the differential posttranslational modifications in the histones at *PRDM1* promoters in the PRDM1β-expressing and non-expressing cell lines. Indicative of promoter activation, similar levels of H3K4me3 and H3K9ac were found in both promoters in U266 cells (PRDM1α positive and PRDM1β positive) and RPMI-8226 cells (PRDM1α positive and PRDM1β negative). However, the enrichment of H3K4me1, H3K9me2, and, to a lesser extent, H3K27me3 was much higher at the *PRDM1β* promoter in the RPMI-8226 cells, suggesting that the abundance of these modifications may regulate the transcriptional activity of the *PRDM1β* promoter towards repression. In general, the H3K4me1 histone mark has been associated with enhancer activity, particularly in B-cell fate^54^, and it may also be possible that the *PRDM1β* promoter acts as an enhancer/promoter region “poised” to be activated while awaiting additional signalling (e.g., acetylation of H3K9).

PRDM1 expression has been considered an oncogene in MM^55^, but in-depth analysis of the differential expression of its isoforms has been lacking, until the present work was performed. We and other researchers have analysed public microarray data comparing normal plasma cells and samples from multiple myeloma, and no correlations were observed for low or high PRDM1 expression levels with the prognosis of the disease^56^. Unfortunately, these analyses were mainly based on microarray data, which were not designed to discriminate between PRDM1α or PRDM1β isoforms, and new analysis data sets (RNA-seq, new microarrays, etc.) comprising possible alternative isoforms are necessary to discern whether differential PRDM1 expression may be related to the pathophysiology of the disease. In conjunction with the transcriptional regulation described in this study, other post-transcriptional regulatory mechanisms involving miRNAs may be key to regulating the expression of PRDM1, as altered expression of some miRNAs targeting PRDM1 mRNA has been described during plasma cell differentiation and in malignant plasma cells^55,57^. Whether these miRNAs specifically modulate the *PRDM1α* or the *PRDM1β* isoform remains to be explored.

**Figure 7:**
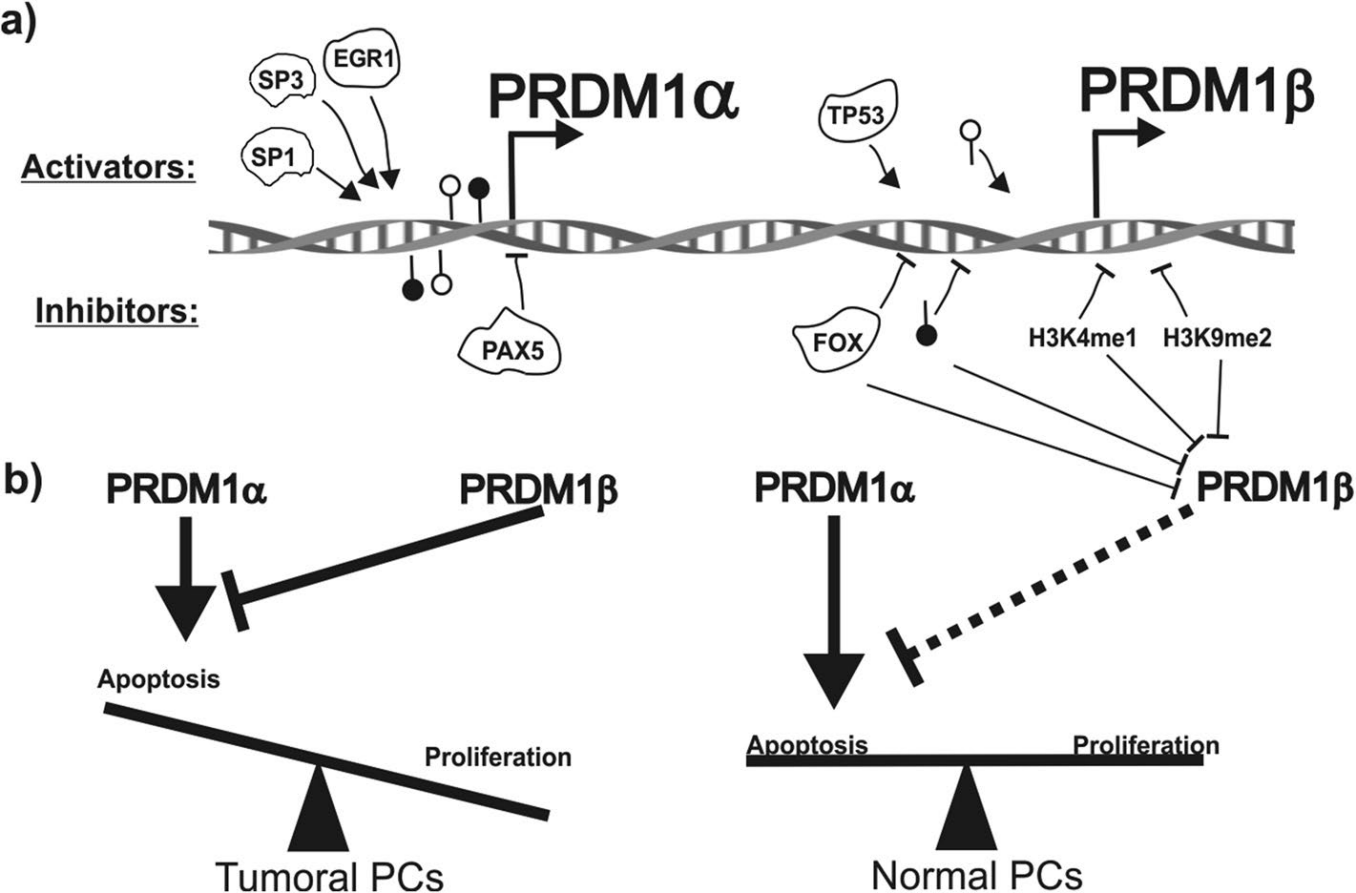
Schematic representation of the regulation of human *PRDM1* promoters in multiple myeloma. **a** Factors such as SP1, SP3 and EGR1 act on the *PRDM1α* promoter, inducing its transcription^36^, the opposite effect as that of PAX5^37^. On the *PRDM1β* promoter, TP53 (as an activator)^41,48^ and FOX factors (as inhibitors) can regulate *PRDM1β* transcription. Regarding epigenetic regulation, methylation seems to play a decisive role in *PRDM1β* expression but not in *PRDM1α* expression. In the case of histone modifications, H3K4me1 and H3K9me2 marks seem to be linked to the repression of the *PRDM1β* promoter. Activators and inhibitors are represented above and under the DNA helix, respectively. Filled and open lollipops represent the methylation and demethylation status of the promoter, respectively. This figure was created using Corel Draw 2018 version 20.1.0.708 software (Corel Corporation, Ottawa, Canada). **b** Schematic representation showing how PRDM1β expression tilts the balance in favour of proliferation due to a diminished apoptosis rate. Inhibiting PRDM1β expression using drugs targeting epigenetic elements at its promoter may restore the balance to maintain normal PC cell fate.

In summary, our results indicated that *PRDM1* gene promoters are modulated through a broad range of genetic and epigenetic control mechanisms that depend on the tumour cell line, supporting further investigation into the transcription and epigenetic factors involved in the direct or indirect regulation of *PRDM1β* transcription and their possible implications in myelomagenesis. In the case of novel treatments of multiple myeloma (Fig. 7), the different transcriptional activity found in the *PRDM1α* and *PRDM1β* promoters in response to treatment with epigenetic modifiers may pave the way to the generation of more specific and tailored epigenetic-based therapeutic strategies for this disease by favouring the expression of PRDM1α over that of PRDM1β.

## Materials and methods

### Human cell lines

The human cell lines U266, NCI-H929, RPMI-8226, HeLa, Daudi and Raji were obtained from The European Collection of Authenticated Cell Cultures (ECACC, Sigma-Aldrich, Madrid, Spain) and maintained in RPMI 1640 medium (Gibco Life Technologies, Carlsbad, CA) supplemented with 10% heat-inactivated foetal bovine serum (FBS), 2 mM glutamine, 100 IU/ml penicillin and 100 μg/ml streptomycin (Gibco) at 37 °C in a humidified atmosphere containing 5% CO_2_ and were routinely verified by morphology and periodically checked for Mycoplasma contamination.

### Patient tissue samples and normal cells

Multiple myeloma PCs (MM-PCs) were purified from donated bone marrow aspirates from patients diagnosed with MM (excluding MGUS or SMM)^58^ using monoclonal antibody anti-CD138-coated microbeads (Miltenyi-Biotec, Auburn, CA, USA). Normal PCs were obtained from discarded bone marrow specimens with no abnormalities at diagnosis after FAC sorting CD138^+^ CD19^+/−^ CD38^+++^ cells. Naïve human B-lymphocytes were isolated from peripheral blood samples from normal volunteers after T-cell depletion by a rosette technique and FAC-sorted to obtain CD19^+^ CD27^−^ IgD^+^ cells. All human samples (MM *n* = 7, normal PCs, *n* = 4, and B-cells, *n* = 3) were obtained from adult donors upon written informed consent under the supervision and approval of the Institutional Review Board (code PI11/01091, date 11/10/2011, Ethics Committee for Research of the Hospital Universitario “Puerta del Mar”, Cadiz, Spain). The Andalusian Public Health System Biobank coordinated the collection, processing, management and assignment of all biological samples used in this study according to standard procedures established for this purpose.

### Reagents

Antibodies were purchased from the following suppliers: anti-H3K9me2, anti-H3K27me3 and anti-H3K9ac (EpiGentek, Farmingdale, NY); anti-H3K4me3, anti-H3K4me1 and anti-β-actin (Abcam, Cambridge, UK); negative rabbit IgG (Cell Signaling Technologies); anti-PRDM1 (sc-66015, Santa Cruz Biotechnologies, Dallas, TX); anti-TP53 (P6874 from Sigma and sc-98 from Santa Cruz); anti-mouse HRP-conjugated and anti-tubulin (Sigma); and anti-FOXA1 from Santa Cruz. Recombinant human TP53 protein (ref. 506165) was obtained from Calbiochem (San Diego, CA). The inhibitor 5-aza-2′-deoxycytidine (5-aza-dC) was purchased from Sigma-Aldrich and dissolved in DMSO.

### Isolation of the 5′ flanking region of the human PRDM1β promoter and luciferase reporter assays

The 5′-flanking region of the human *PRDM1* gene (− 2077/+ 41) was isolated by PCR. Two specific primers, PRDM1β-BamHI-F (forward, − 2077 bp relative to the translation start site including a restriction site for the BamHI enzyme) and PRDM1β-BglII-R (reverse, + 41 bp relative to the translation start site including a part of exon-1β and a restriction site for the BglII enzyme) (Table 1), were selected on the basis of the sequence of the human *PRDM1* genomic DNA accession number AL022067. The resulting PCR fragment was digested with BamHI/BglII and cloned into a pGL3-basic firefly luciferase reporter vector (Promega, Madison, WI) at the BglII site to obtain the pGL3-BamHI construct. This construct was digested with the NheI/XbaI restriction enzymes to obtain the pGL3-XbaI construct, which was subsequently digested with the KpnI restriction enzyme to obtain the pGL3-KpnI construct. The pGL3-S1, pGL3-S2, pGL3-S3 and pGL3-S4 constructs were generated by PCR using the following forward oligonucleotide primers, respectively, in Table 1: PRDM1β-S1-F, PRDM1β-S2-F, PRDM1β-S3-F and PRDM1β-S4-F (all including a restriction site for the enzyme SmaI at the 5′ ends) together with the PRDM1β-BglII-R oligonucleotide as the reverse primer (Table 1). The resulting PCR products were digested with SmaI/BglII restriction enzymes and cloned into a pGL3-basic plasmid. Primary sequences of the inserts were validated by Sanger sequencing. All *PRDM1β* promoter constructs were transfected into U266 cells using the Nucleofector Kit C Program X-005 with an Amaxa nucleofector system following the manufacturer’s instructions (Lonza, Barcelona, Spain) or into NCI-H929 cells using a Gene Pulser II apparatus (Bio-Rad, Madrid, Spain) set at 400 µF and 240 V, with a time constant of t ~ 20 ms. The cells were harvested after 48 h, and luciferase activity was measured using a dual luciferase reporter assay kit (Promega). Each transfection experiment was performed in triplicate with at least two different plasmid preparations.

**Table 1:** Oligonucleotide sequences used for the different assays.

**Cloning reporter constructs**		
PRDM1β-BamHI F		ctcgaggatccGTGCATACTTACATGAAATG
PRDM1β-S1 F		tcccgGGTGTTTTAGCCAGCCTCCT
PRDM1β-S2 F		tcccgggTGTCAAATTCGGGCTGCTGC
PRDM1β-S3 F		tcccggGTGTAGGAAACGGCGAGTACAG
PRDM1β-S4 F		tcccgggCTACCCAGTGACTCAAAGCAC
PRDM1β-BglII R		tctagatctTTTGCTACTGCAGTGAATGGC
**ChIP-qPCR**		
PRDM1α F		GGAAGCCAGACGGTTAAC
PRDM1α R		CTCGGCGGTCCCTCCTCG
PRDM1β F		GTGTAGGAAACGGCGAGTACAG
PRDM1β R		AGATCTTTTCCATTTCAGTTTTATCGTCTTTTCATGTTCG
**Bisulfite sequencing nested PCR**		
PRDM1β 1F		GGTTTTAAGTATTTATATGAATAAGTGTTATTTTAGG
PRDM1β 1R		CCTACTACCTAATTAAAAAAAAAATCCAATC
PRDM1β 2F		TTGGGGGTGGAGGATGTTTGTATAGTTG
PRDM1β 2R		ATCCAATCAGTTTAGCCAGAAT
PRDM1α 1F		AGAATATAATAGAGTTTAAGTAAGTGTTGAGG
PRDM1α 1R		AAATTTTCAAATCAATTTCAAAATTA
PRDM1α 2F		AAGTGTTTTTAAAGGGAAGTAAGAAG
PRDM1α 2R		CCAAACAAATATCCAACATCTAAAAAAAATCTC
**Transcripts expression by RT-qPCR**		
PRDM1α exon 3–4	Probe	FAM/TGGAGGATC/ZEN/TATTCCAGAGGGGAGC/IABkFQ
	*Primer* 1	CCTAAGAACGCCAACAGGAA
	*Primer* 2	AGAGTGTGCTGGATTCACATAG
PRDM1β exon 1β-4	Probe	FAM/CCCTCTGGA/ZEN/ATAGGATCTTTTCCATTTC/IABfQ
	*Primer* 1	TTGAGGCAGCTCCTTAAATG
	*Primer* 2	CATTAAAGCCGTCAATGAAGTG
β-actin	Probe	FAM/TCATCCATG/ZEN/GTGAGCTGGCGG/IABfQ
	*Primer* 1	ACAGAGCCTCGCCTTTG
	*Primer* 2	CCTTGCACATGCCGGAG
**EMSA probes**		
wtFOXA1 strand+		GGAAAGTGCAAGTCTGGACATGTTTATCAAGAGG
wtFOXA1 strand−		CCTCTTGATAAACATGTCCAGACTTGCACTTTC
mutFOXA1 strand+		GGAAAGTGCAAGTCTGGACATG**cc**TATCAAGAGG
mutFOXA1 strand−		CCTCTTGATAggCATGTCCAGACTTGCACTTTC
TP53 strand+		TACAGAACATGTCTAAGCATGCTGGGG
TP53 strand−		GCCCCAGCATGCTTAGACATGTTCTGTA

All sequences are shown from 5′ → 3′. Lowercase letters indicate endonuclease restriction sites just intended for cloning, or just in case of probe mutFOXA1 for EMSA indicate the nucleotides mutated. FAM is fluorescein amidite fluorophore; ZEN is IDT’s ZEN dark quencher; IABFQ is IDT’s Iowa Black FQ dark quencher.

### Chromatin immunoprecipitation assays

The chromatin immunoprecipitation (ChIP) procedure was performed using a SimpleChIP Enzymatic Chromatin IP Kit as recommended in the manufacturer’s instructions (Cell Signaling Technologies, Barcelona, Spain). Briefly, RPMI-8226 cells or U266 cells (40 × 10^6^) were incubated with 1% formaldehyde for 10 min at room temperature. Chromatin was sheared to an average size of 200 to 1000 bp by enzymatic digestion and sonication with a Sonifier-150 microtip probe (Branson Ultrasonics, Danbury, CT) using the following parameters: 1 pulse × 15 s, output control 2.5 for U266 cells and 2 pulses × 15 s, output control 2 for RPMI-8226 cells maintained on ice for 30 s between pulses. After a spinning step to reduce debris, immunoprecipitated cross-linked complexes were prepared using 3 µg of the following antibodies specific for histone modifications: anti-H3K9me2 and anti-H3K9ac (EpiGentek, Farmingdale, NY), anti-H3K4me3 and anti-H3K4me1 (Abcam, Cambridge, UK) and negative rabbit IgG (Cell Signaling Technologies). After an incubation overnight at 4 °C on a rocking platform, 30 μl of ChIP-grade protein G agarose beads were added and incubated for 2 h at 4 °C with rotation. Then, the bound antibody-protein-DNA complexes were washed, and after cross-linking reversal and proteinase K digestion, each individual immunoprecipitated complex was purified using spin columns. Real-time PCR was performed with the corresponding specific primers indicated in Table 1.

### Promoter methylation analysis

The methylation of the *PRDM1α* and *PRDM1β* promoters was assessed by bisulfite sequencing. Genomic DNA was extracted from all cell types analysed using a ZR-Duet DNA/RNA Miniprep kit (Zymo Research, Irvine CA), and then, 500 ng of DNA was used for the bisulfite modification with a premium bisulfite kit according to the manufacturer’s instructions (Diagenode, Belgium). Bisulfite sequencing nested-PCR was performed to assess the methylation of the CpG islands in the *PRDM1α* and *PRDM1β* promoters using *PRDM1α* and *PRDM1β* bisulfite-specific primers (Table 1) and Q5 High-Fidelity 2X Master Mix (New England Biolabs, Ipswich, MA). The amplified DNA was cloned into a pSpark I vector (Canvax, Córdoba, Spain), and more than 10 individual clones were Sanger sequenced. The obtained sequences were analysed by the online BISMA (bisulfite sequencing DNA methylation analysis) program^59^.

### 5-Aza-dC treatment

RPMI-8226, U266, NCI-H929 or MM-isolated cells were cultured with or without the inhibitor 5-aza-2′-deoxycytidine (5-aza-dC; 0.5, 1, or 2 µM) (Sigma), which was initially dissolved in DMSO. The cells were cultured at 37 °C in a 5% CO_2_ incubator for 48 h. After incubation, the viability of the cells was measured using Trypan blue, and the variations in the expression of the *PRDM1* transcript isoforms were studied by RT-qPCR. All reactions were performed with identical conditions in triplicate.

### Retrotranscription and RT-qPCR

For the *PRDM1α* and *PRDM1β* transcript analysis, the total RNA from the cultured cells was purified using NucleoSpin RNA XS (Macherey–Nagel, Düren, Germany) and reverse transcribed using random hexamers with a Maxima H Minus First Strand cDNA synthesis kit (Thermo Fisher, Waltham, MA). Then, the cDNAs were assessed with real-time PCR TaqMan probes for *PRDM1α*, *PRDM1β* and *β-actin* (Table 1) and a SensiFAST Probe No-ROX kit (Bioline, London, UK) in a RotorGene 6000 (Corbett, Sidney, Australia). The relative quantitative values were calculated using the 2^−ΔΔCT^ method^60^.

### Western blotting

Western blotting was performed previously described^61^. Fifty micrograms of total protein lysates were added to each well. The following antibodies were used at the indicated dilutions: anti-PRDM1 (sc-66015 from Santa Cruz Biotechnologies, Dallas, TX), anti-β-actin (ab6276 from Abcam), anti-TP53 (P6874) and anti-mouse HRP-conjugated (A2554 from Sigma). The bound antibodies were detected using a WesternBright Sirius kit (Advansta, Menlo Park, CA) and a ChemiDoc XRS system (Bio-Rad, Madrid, Spain). As indicated, the blots were reprobed with the β-actin or α-tubulin antibody as a loading control.

### Electrophoretic mobility shift assays (EMSAs)

Nuclear extracts (NEs) were prepared from the cell by hypotonic lysis, followed by high salt extraction of nuclei as previously described^62^. All probes and cold fragments used for the competition assays (Table 1) were obtained by annealing synthesized complementary oligonucleotides with protruding ends and labelled by Klenow filling with [*α*-^32^P]-dCTP (Perkin-Elmer, Madrid, Spain). The DNA–protein-binding assays were carried out as previously described^37^. For the competition studies, 100-fold molar excess competitor DNA was added to the reaction mixture 10 min prior to the labelled probe. Supershift assays were performed by preincubating the NEs with an antibody against TP53 purchased from Sigma (P6874) or Santa Cruz (sc-98) as indicated or anti-FOXA1 purchased from Santa Cruz (sc-6553) for 1 h on ice before the probe addition. As indicated, recombinant human TP53 protein (ref. 506165) from Calbiochem (San Diego, CA) was used instead of the nuclear extracts.

### Apoptosis and cell proliferation assays

U266, RPMI-8226 and NCI-H929 cells were transfected with a pIRES2-EGFP empty vector (Clontech, Takara Bio USA Inc, USA) or a vector containing the cDNAs for PRDM1α or PRDM1β, both with an HA epitope. The cells were harvested after 48 h and treated for apoptosis or proliferation analysis. Apoptosis was determined by Annexin V-allophycocyanin (APC) staining according to the protocol of the manufacturer (ImmunoTools, Friesoythe; Germany). Cell proliferation was determined with a Click-iT plus Edu Alexa 647 flow cytometry assay kit following the manufacturer’s instructions (Invitrogen, Carlsbad, CA). Only EGFP^+^-transfected cells were selected for apoptosis and proliferation analyses by a FACSCalibur flow cytometer with CellQuest software (Becton Dickinson, Franklin Lakes, NJ). To validate the transfections, Western blotting was performed as described above using a conjugate HRP antibody against the HA epitope (ref. A00169, GenScript, Piscataway, NJ).

### Statistical analysis

The statistical package SPSS version 21 was used. All variables exhibited a nonparametric distribution after applying the Kolmogorov–Smirnov test; thus, the data were analysed using the nonparametric Mann–Whitney U test (two sided) to determine significant differences between two experimental groups. The sample size (*n*) for each experimental group/condition is given in the corresponding figure legend. The results are generally expressed as the means ± SEM, and *p* < 0.05 was considered statistically significant.

## Supplementary information

Supplementary information

Supplementary figures
