# Supplementary material for: Differential epigenetic regulation between the alternative promoters, PRDM1α and PRDM1β, of the tumour suppressor gene PRDM1 in human multiple myeloma cells
Source: Sci Rep. 2020 Sep 28;10:15899. doi: 10.1038/s41598-020-72946-z (PMC7522722; doi:10.1038/s41598-020-72946-z)

**Supplementary Information: Full length gel/blot images**

**Differential epigenetic regulation between the alternative promoters, *PRDM1α* and *PRDM1β*, of the tumour suppressor gene *PRDM1* in human multiple myeloma cells**

Raquel Romero-García, Laura Gómez-Jaramillo, Rosa María Mateos-Bernal, Gema Jiménez-Gómez, Nuria Pedreño-Horrillo, Esther Foncubierta, Juan Francisco Rodríguez-Gutiérrez, Sebastián Garzón, Francisco Mora-López, Luis M. Valor, and Antonio Campos-Caro*

**Full length EMSA shown in Figure 3**


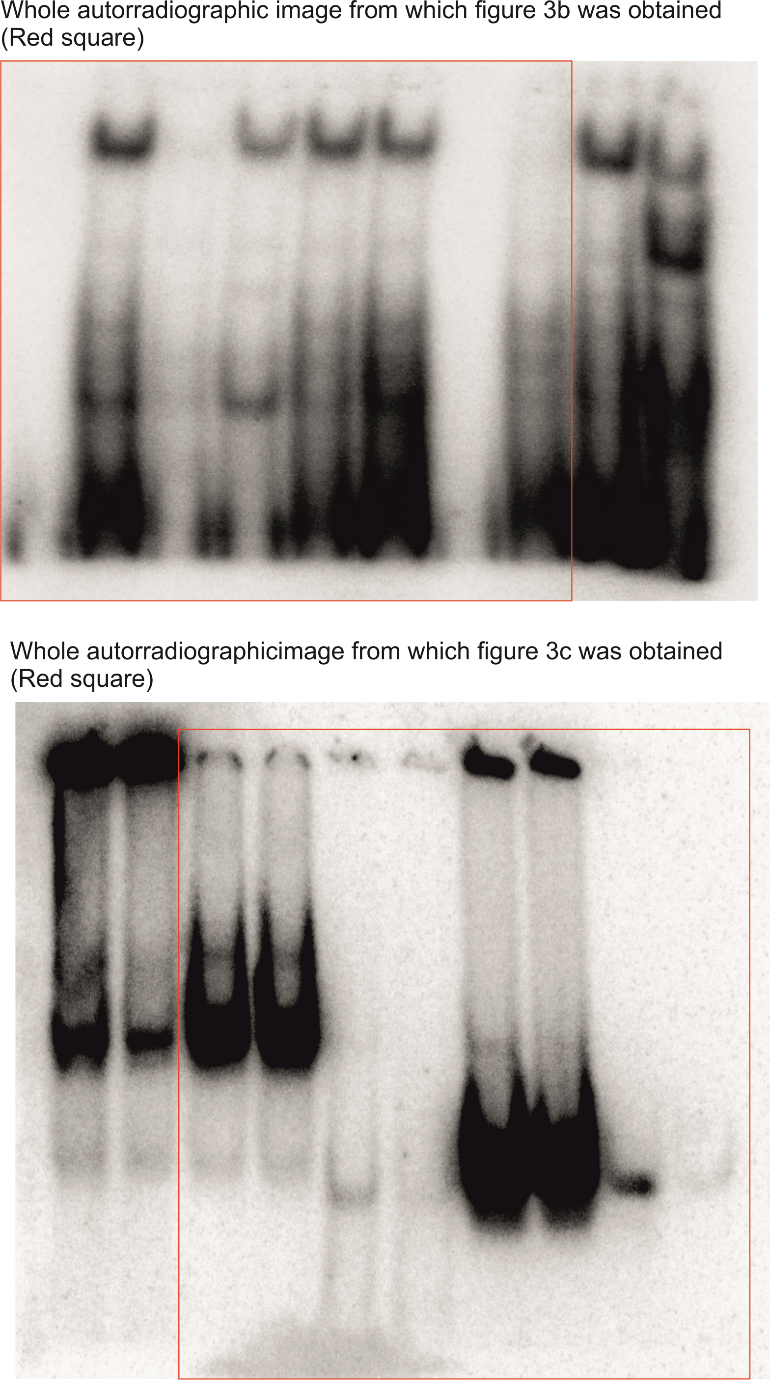


**Full length blots for figure 4c**


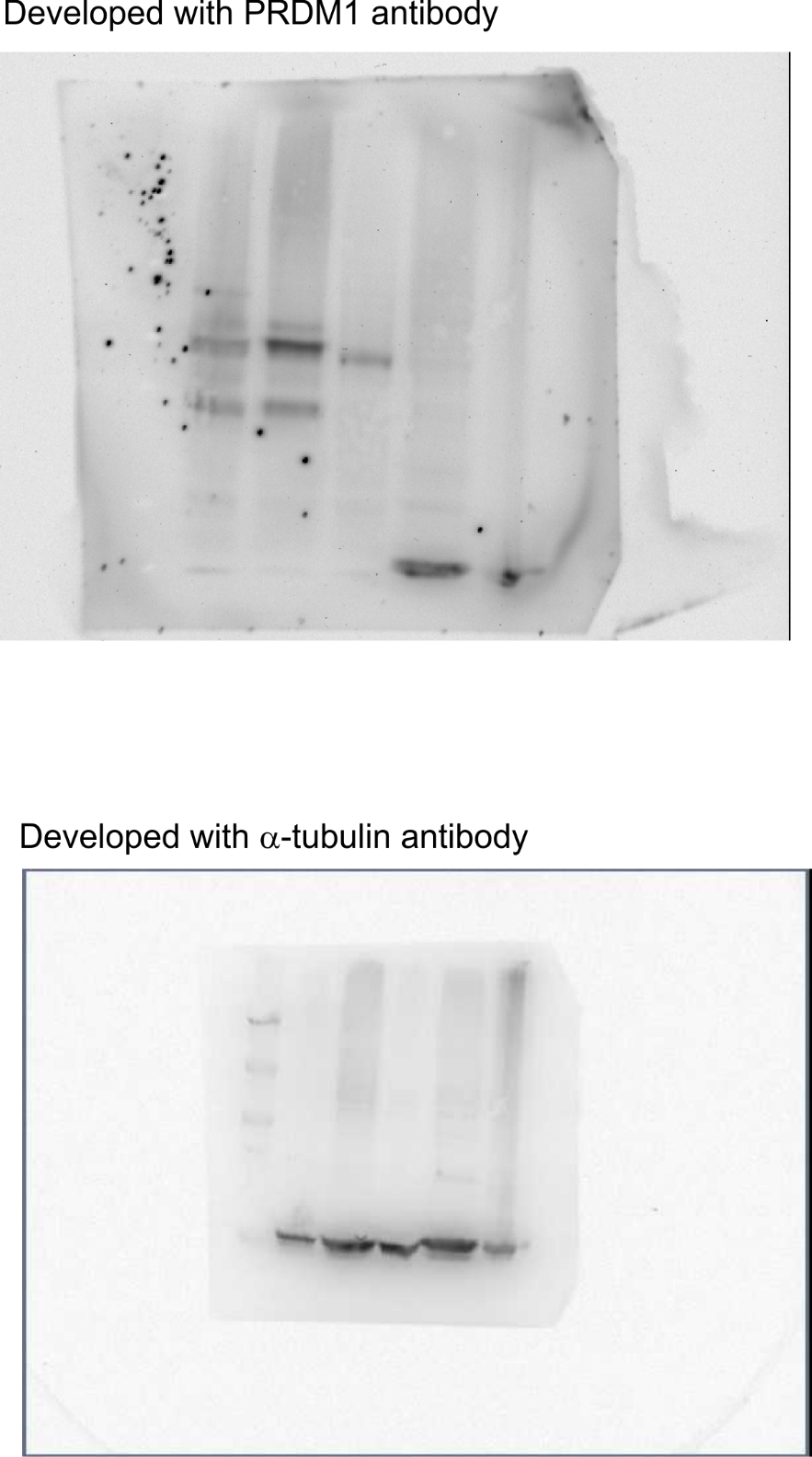

Supplement: Supplementary file 1 — Supplementary information [file 41598_2020_72946_MOESM1_ESM.docx]
