# Supplementary material for: Differential epigenetic regulation between the alternative promoters, PRDM1α and PRDM1β, of the tumour suppressor gene PRDM1 in human multiple myeloma cells
Source: Sci Rep. 2020 Sep 28;10:15899. doi: 10.1038/s41598-020-72946-z (PMC7522722; doi:10.1038/s41598-020-72946-z)
Supplement: Supplementary file 2 — Supplementary figures [file 41598_2020_72946_MOESM2_ESM.docx]

**Supplementary Information**

**Differential epigenetic regulation between the alternative promoters, *PRDM1α* and *PRDM1β*, of the tumour suppressor gene *PRDM1* in human multiple myeloma cells**

Raquel Romero-García, Laura Gómez-Jaramillo, Rosa María Mateos-Bernal, Gema Jiménez-Gómez, Nuria Pedreño-Horrillo, Esther Foncubierta, Juan Francisco Rodríguez-Gutiérrez, Sebastián Garzón, Francisco Mora-López, Carmen Rodríguez, Luis M. Valor, and Antonio Campos-Caro*


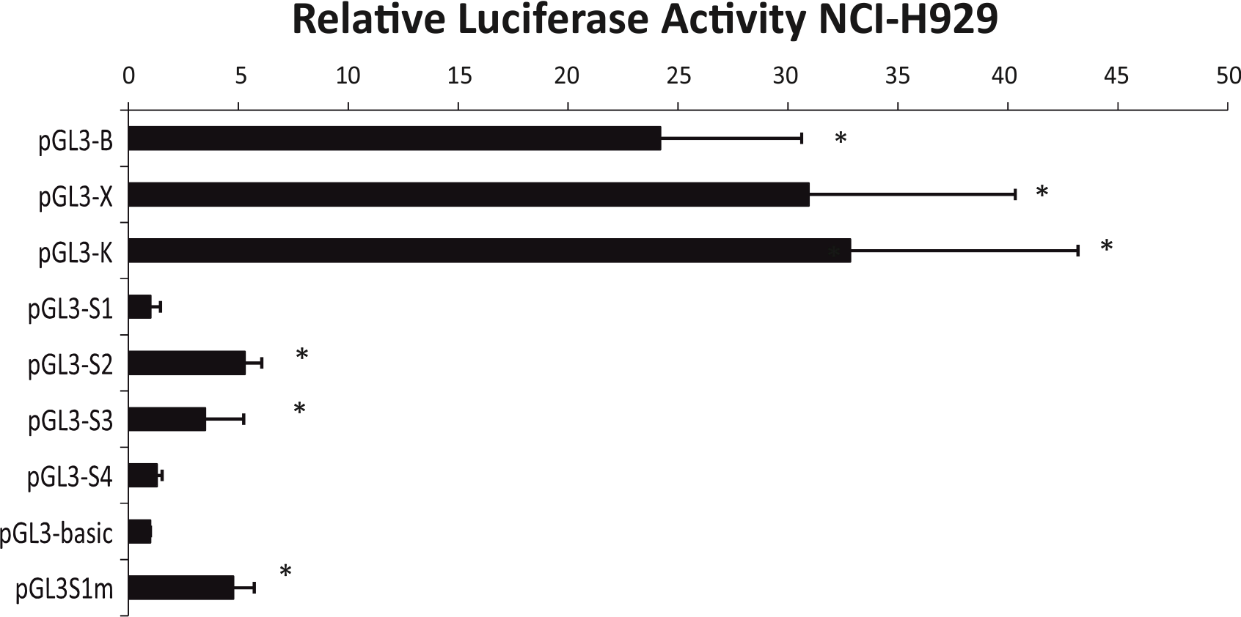


**Figure S1.** **Relative luciferase activities of the *PRDM1β* promoter constructs in NCI-H929 cell line.**


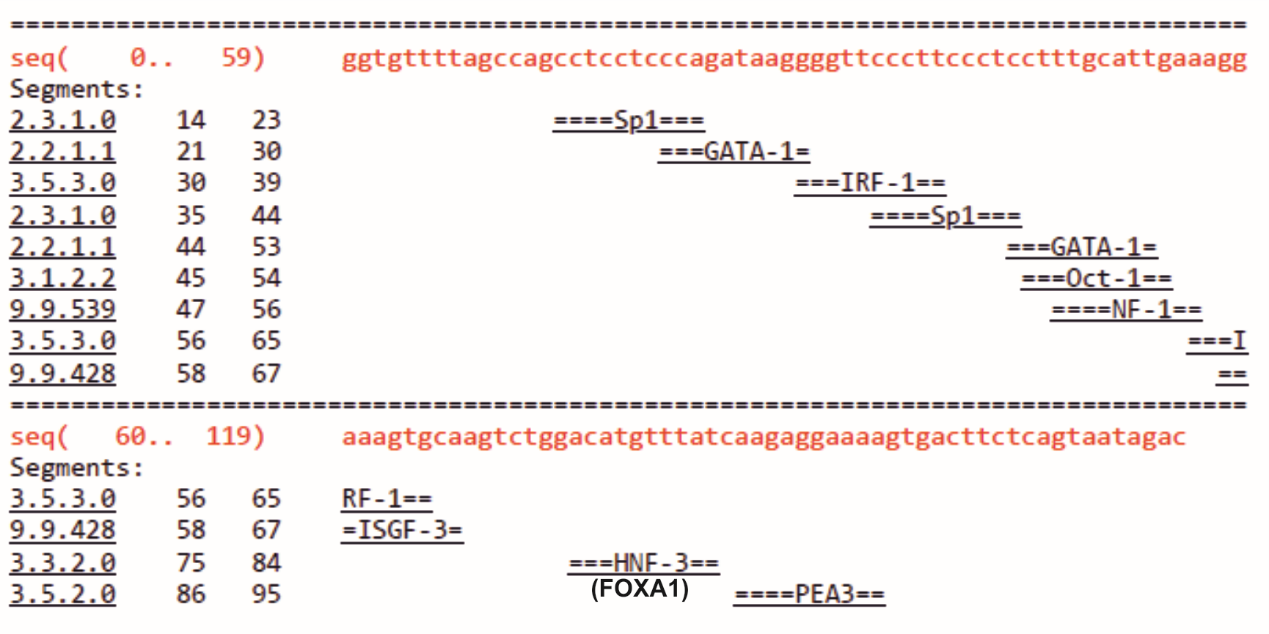


**Figure S2.** ***In silico* analysis of the sequence corresponding to the region flanked by the S1 and S2 fragments.** In addition to the FOXA1 (HNF3) binding site, other common transcriptional factors were identified (SP1, GATA1, IRF1, etc.).


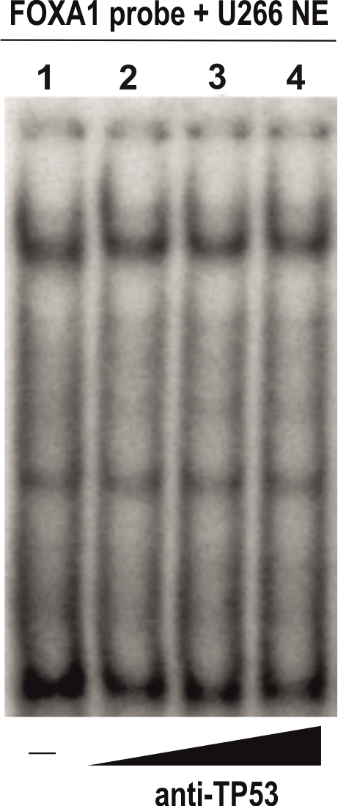


**Figure S3. Super-shift EMSA assay with anti-TP53 antibody.** U266 NE were incubated with the wtFOXA1 probe (lane1). In addition to U266 NE, lanes 2-4 were incubated with increasing amounts (0.5, 1 and 2 μg) of anti-TP53 antibody (sc-98, Santa Cruz). No difference was observed compared to the control (lane 1) in any retarded bands.


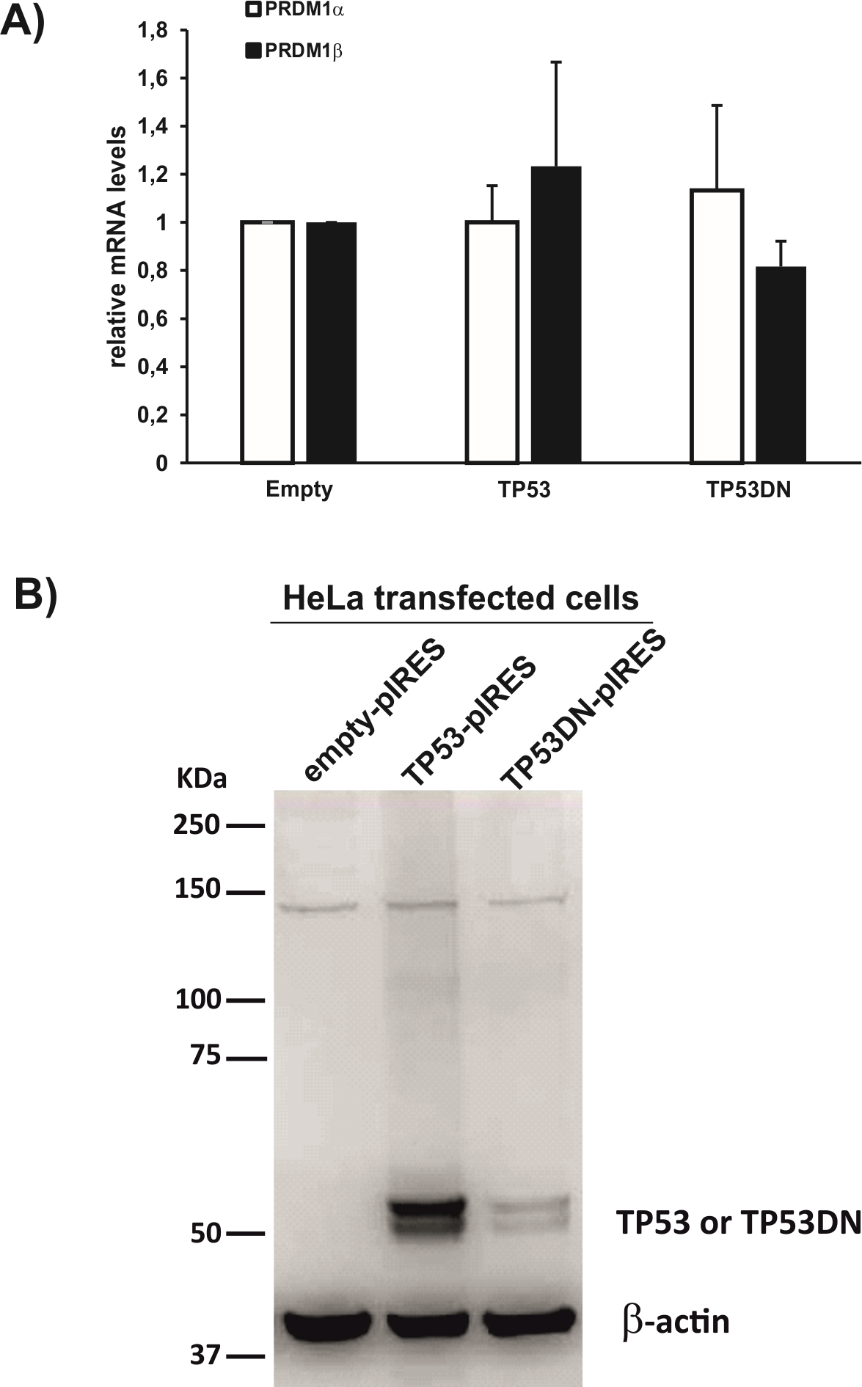


**Figure S4.** **Exogenous expression of human TP53 does not alter the *PRDM1α* and *PRDM1β* transcript levels in HeLa cells.** HeLa cells were transfected with the plasmid vector pIRES-2EGFP (Clontech, Mountain View, CA), either empty as a control or bearing the cDNA coding wild-type TP53 or a dominant negative mutant TP53 (TP53DN). Then, 48-72 h after transfection, the cells were isolated by a BD FACS Aria sorter (Becton Dickinson, NJ) based on the EGFP expression, and the cell pellets were split and processed in parallel for **a** RNA isolation and cDNA synthesis intended for *PRDM1α* and *PRDM1β* transcript analysis by RT-qPCR. Despite the expression of exogenous TP53 and TP53DN in HeLa cells, the basal expression of the *PRDM1α* and *PRDM1β* transcripts did not significantly change (n=3), and **b** a representative TP53 expression analysis by western blotting using an anti-TP53 antibody (ref. P6874, Sigma) is shown.


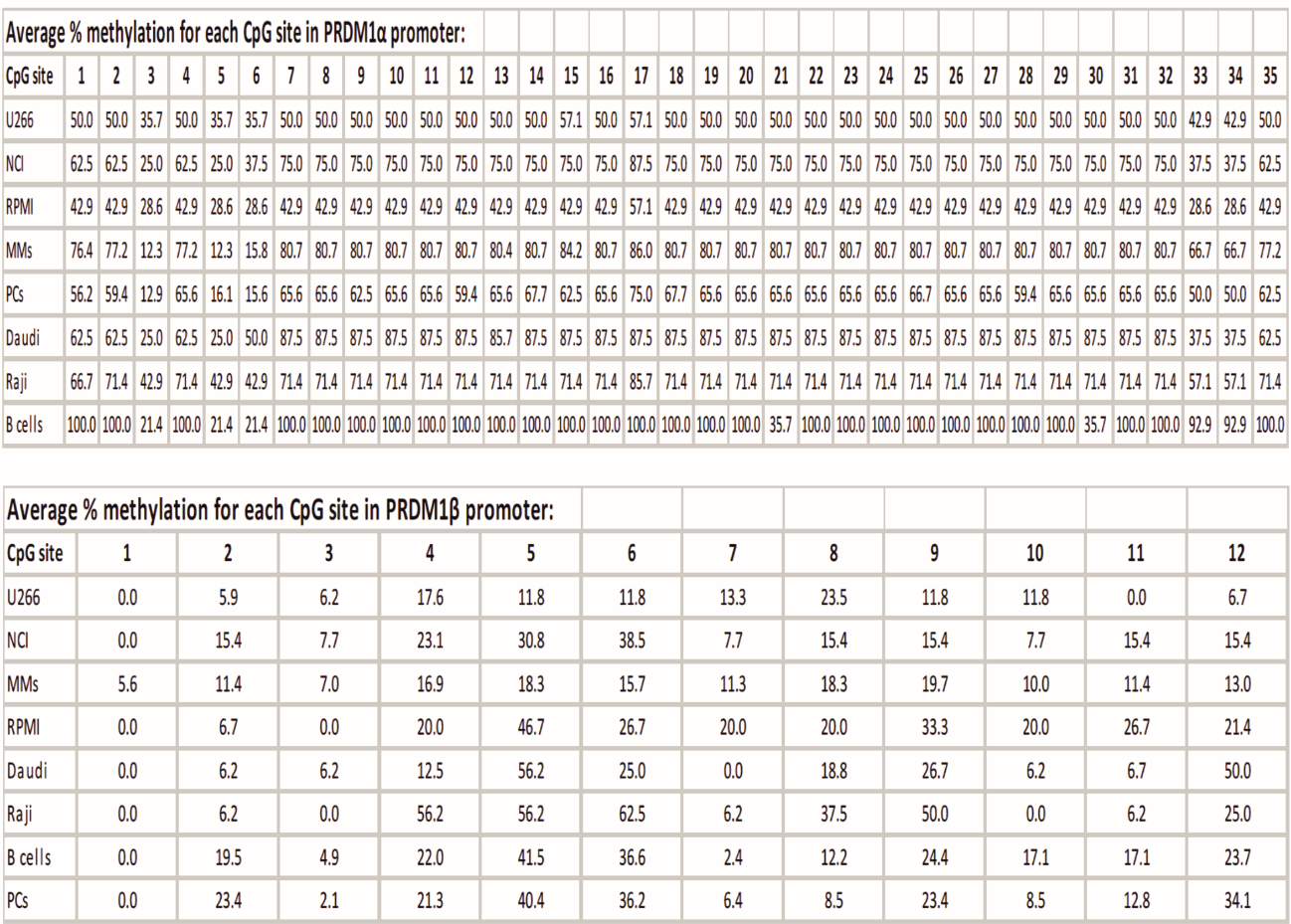


**Figure S5. Methylation analysis of *PRDM1α* and *PRDM1β* promoters in normal PCs and B-cells, cell lines and multiple myeloma primary tumours.** The percentage of methylation determined by bisulfite cloning is summarized at each CpG position in all cell subsets.
